# Stillbirths, Neonatal Morbidity, and Mortality in Health-Facility Deliveries in Urban Gambia

**DOI:** 10.3389/fped.2021.579922

**Published:** 2021-02-15

**Authors:** Bully Camara, Claire Oluwalana, Reiko Miyahara, Alyson Lush, Beate Kampmann, Kebba Manneh, Uduak Okomo, Umberto D'Alessandro, Anna Roca

**Affiliations:** ^1^Medical Research Council Unit the Gambia at London School of Hygiene and Tropical Medicine, Banjul, Gambia; ^2^Department of Clinical Tropical Medicine, Institute of Tropical Medicine, Graduate School of Tropical Medicine, Nagasaki University, Nagasaki, Japan; ^3^Bundung Maternal and Child Health Hospital, Banjul, Gambia

**Keywords:** stillbirth, neonatal mortality, congenital malformation, sepsis, birth asphyxia, hospitalization, The Gambia

## Abstract

**Background:** The Gambia Demographic and Health Survey 2013 data showed that up to 63% of deliveries in the country occur in health facilities. Despite such a high rate, there are few facility-based studies on delivery outcomes in the country. This analysis ancillary to a randomized control trial describes occurrence of poor pregnancy outcomes in a cohort of women and their infants delivering in a government health facility in urban Gambia.

**Methods:** Using clinical information obtained during the trial, we calculated rates of poor pregnancy outcomes including stillbirths, hospitalization and neonatal deaths. Logistic regression was used to calculate odds ratio (OR) and 95% confidence interval (CI) in the risk factors analysis.

**Results:** Between April 2013 and 2014, 829 mothers delivered 843 babies, including 13 stillbirths [15.4 (7.1–23.8)] per 1,000 births. Among 830 live born infants, 7.6% (*n* = 63) required hospitalization during the 8-week follow-up period. Most of these hospitalizations (74.6%) occurred during the early neonatal period (<7 days of life). Severe clinical infections (i.e., sepsis, meningitis and pneumonia) (*n* = 27) were the most common diagnoses, followed by birth asphyxia (*n* = 13), major congenital malformations (*n* = 10), jaundice (*n* = 6) and low birth weight (*n* = 5). There were sixteen neonatal deaths, most of which also occurred during the early neonatal period. Overall, neonatal mortality rate (NMR) and perinatal mortality rate (PMR) were 19.3 (CI: 9.9–28.7) per 1,000 live births and 26.1 (CI: 15.3–36.9) per 1,000 total births, respectively. Severe clinical infections and birth asphyxia accounted for 37 and 31% of neonatal deaths, respectively. The risk of hospitalization was higher among neonates with severe congenital malformations, low birth weight, twin deliveries, and those born by cesarean section. Risk of mortality was higher among neonates with severe congenital malformations and twin deliveries.

**Conclusion:** Neonatal hospitalization and deaths in our cohort were high. Although vertical interventions may reduce specific causes of morbidity and mortality, data indicate the need for a holistic approach to significantly improve the rates of poor pregnancy outcomes. Critically, a focus on decreasing the high rate of stillbirths is warranted.

**Clinical Trial Registration:** ClinicalTrials.gov Identifier: NCT01800942.

## Introduction

Newborn health is a global health priority due to unacceptable high levels of morbidity and mortality in children <1 month old (1). Almost half of deaths in children occur during the neonatal period (2), and 3 out of 4 neonatal deaths occur within the first week of life (3). While the decline in neonatal mortality has consistently lagged behind that of infants and older children, the number of stillbirths has reduced even more slowly over the years (4). It is estimated that there are up to 2.6 million stillbirths each year, with over 75% of deaths occurring during labor, primarily in low income countries (4). Sub-Saharan Africa (SSA) and Southern Asia both account for almost 80% of the annual global burden of perinatal deaths (2).

Globally, the major causes of neonatal morbidity and mortality are prematurity, intrapartum related complications, severe infections and congenital malformations (5). The distribution of cause of death differs throughout the neonatal period (early vs. late neonatal period) and by neonatal mortality rate (3). In general, infections and intrapartum-related events account for a higher proportion of deaths in higher mortality settings (3). Risk factors associated with poor pregnancy outcomes include limited access to skilled care during delivery and poor socio-economic conditions (6), both common in SSA, where the poorest households carry the greatest burden (7, 8). Poor maternal nutrition and multiple-pregnancy, both associated with prematurity and low birth weight, are also indirect causes of poor pregnancy outcomes (9). Additionally, it is difficult to accurately quantify the rates of poor outcomes in SSA, even in health facilities, as death certificates are often not issued for stillbirths, making it difficult to quantify the burden of stillbirths in the region (4). For neonatal deaths outside of health facilities, cause of death is mostly established by verbal autopsy, which can be inaccurate (10). Despite this, and although the main cause of neonatal death differs by country and geographical regions (7, 8), many deaths can be prevented if the risk is recognized early to implement appropriate treatment (11, 12).

The Sustainable Development Goal 3, Target 3.2 is to decrease neonatal mortality to <12 per 100,000 live-births by 2030 in all countries (13). In general, data on neonatal outcomes in West Africa is scarce. In The Gambia, although the mortality rate for under-5s has decreased by more than two-thirds in the last 10 years, reduction of the neonatal mortality rate has occurred at a slower pace and recent data on the rate of stillbirths in the country is not available (14). In this study, we assessed rates of stillbirths, neonatal and perinatal morbidity and mortality rates in a cohort of infants born to 829 mothers in a busy urban health facility in western Gambia. Women were originally recruited into a randomized control trial and were followed, with their children, for a period of 8 weeks after delivery to determine neonatal and perinatal outcomes (15).

## Methods

### Main Trial

This is a secondary analysis of the PregnAnZI trial (NCT01800942 at ClinicalTrials.gov), a phase-III, double-blind, placebo-controlled individually randomized clinical trial in which 829 women in labor were allocated to receive either a single dose of oral azithromycin or placebo (ratio 1:1). The study results showed that there were differences in bacterial colonization and non-serious clinical conditions between study arms. However, the study was not powered to determine differences in severe neonatal morbidity and mortality, and therefore, we have included the full trial cohort in our analysis presented here (16).

### Study Setting

The study was based at the Bundung Maternal and Child Health Hospital, previously Jammeh Foundation for Peace (JFP) health center, a government-run facility located in western Gambia that manages up to 5,000 deliveries per year (16). The population of the catchment area is representative of The Gambia and includes all main ethnic groups. Illiteracy rates are high, up to 55% among women (14). The level of consanguinity is moderately high at ~30% frequency of first-cousin marriages (17). The climate of the area is typical of the sub-Sahel region (16).

### Study Design and Participants

The trial recruited women aged 18–45 attending antenatal, natal and postnatal care at the study health facility, and their children (16). Women were excluded from participating in the trial if they had an HIV infection, or a chronic or acute condition which might interfere with the study as judged by the research clinicians (15). Women were also excluded if they had a planned cesarean section, known required referral, known multiple pregnancy, known severe congenital malformation of the unborn baby, intrauterine fetal death before randomization, known allergy to macrolides and/or intake of antibiotics in the week before randomization. Mothers who were planning to travel out of the catchment area during the 2 month follow-up period were also excluded (16).

### Study Procedures

The field team conducted sensitization and consenting for women during antenatal clinic visits. Women who presented at the study health facility for delivery were screened to confirm consent and ensure all eligibility criteria were met (16). If a woman who had signed consent was still willing to participate in the study, she was assigned a randomization number and administered the trial investigational product. At the time of the study, the health facility lacked the capacity to provide emergency obstetric care, including surgical care. Women in need of an emergency cesarean section were referred to the Edward Francis Small Teaching Hospital in Banjul, ~20 kilometers from the study site (16). As per study procedures, stillbirth information and birth weight were collected after delivery (16). A thorough clinical assessment of the mother and the baby was carried out by either the trial research clinician or pediatrician 4–24 h after childbirth (15). Women and their babies were visited daily at home by trained nurses for a week post-delivery. After this period, mothers were scheduled to report to the study team at the health facility between days 8 and 13, where a study clinician or pediatrician carried out a thorough clinical assessment of the mother/child pair (16). Field workers visited the mothers and newborns each week for 8-weeks post-partum. Weekly active follow-up visits were complemented with passive follow-up visits at the study health facility and the Clinical Services Department at the Medical Research Council Unit The Gambia (16).

Adverse events were monitored and assessed throughout the follow up period to evaluate safety of the intervention on mothers and newborns (16). Diagnoses of serious infections, perinatal asphyxia and congenital malformation among others were primarily based on clinical judgment of the study clinician and pediatrician (18).

### Data Management and Statistical Analysis

Case report forms were reviewed for accuracy, consistency and completeness before being entered in an OpenClinica (www.openclinica.com) database. Consistency checks and data validation were performed at regular intervals (16).

For this analysis, the neonatal period was defined as 0–56 days after delivery rather than the conventional definition of 0–28 days, as per the study active follow-up period (19). The early neonatal period was defined as 0–7 days, and the late neonatal period 8–56 days after delivery (16).

A stillbirth was defined as the death of a fetus with birth weight ≥0.5 kg or estimated gestation ≥22 weeks (20). Based on clinical assessment, stillbirths were categorized as macerated if the stillborn had any of the following skin changes: slipping skin, bullae formation or desquamation (21). A stillbirth was considered a fresh stillbirth if the skin changes for maceration were absent (21). A congenital malformation was considered “major” if it was life threatening or required surgical correction. Low birth weight was defined as birth weight of <2.5 kilograms (20). If a child had more than one diagnosis during admission, only the main diagnosis was considered in the analysis (15, 16).

Rates of stillbirths were calculated as the number of deaths per 1,000 births. Mortality rates as the number of deaths per 1,000 live births. Perinatal mortality rate (PMR) was calculated as number of stillbirths and deaths in the first week of life (early neonatal period) per 1,000 births (19).

To calculate odds ratios (OR) and 95% confidence intervals (CI) to estimate related risk factors to deaths (including stillbirths) and hospital admissions, we used a logistic regression analysis. Significance was considered when *p* < 0.050. However, variables with *p* < 0.100 in the univariate analysis and sex (defined *a priori* as a potential confounder) were included in the multivariate analysis.

### Ethical Approval

The main trial was approved by the Joint Gambia Government/MRC Ethics Committee (EC). Written informed consent was obtained from mothers prior to delivery and enrolment in the clinical trial during their pregnancy.

## Results

### Maternal and Newborn Baseline Characteristics

We recruited 829 women (median age of 25.5; IQR 22.0, 30.0) who delivered 843 babies. Mandinka (42.8%) was the most common ethnic group. There were 179 (21.7%) primigravidae (Table 1). No maternal deaths were reported during the 8-week follow up period. There were 14 (3.3%) twin deliveries (28 newborns), 55 (6.5%) babies were low birth weight and 10 (1.3%) had major congenital malformations (Table 1). Deliveries were seasonal with peaks observed from September 2013 to March 2014.

**Table 1:** Baseline characteristics of mothers (*N* = 829) and their newborns (*N* = 843).

**Maternal characteristics**	**Freq. *n* (%)**
^*^Age in years	
≤19	67 (8.1)
20–34	701 (85.1)
≥35	56 (6.8)
**Mode of delivery**	
Vaginal	827 (98.1)
Cesarean section	16 (1.9)
**Season of delivery**	
Rainy season	180 (21.4)
Early dry season	165 (19.6)
Late dry season	498 (59.1)
^*^Ethnicity	
Mandinka	352 (42.8)
Fula	144 (17.5)
Jola	127 (15.4)
Wollof	94 (11.4)
Others	106 (12.9)
^*^Occupation	
Yes	128 (15.5)
No	698 (84.5)
^*^Education in years	
<6	467 (56.8)
≥6	333 (40.5)
Unknown	22 (2.7)
^*^Number of children	
1	179 (21.7)
2–3	337 (40.1)
≥4	310 (37.5)
**Newborn Characteristics**	
**Sex**	
Male	438 (52.0)
Female	405 (48.0)
**Gestational age in weeks at birth**	
Pre-term (<37)	473 (56.3)
Term (≥37)	367 (43.7)
**Birth weight in grams**	
Normal (≥2,500)	786 (93.5)
Low(<2,500)	55 (6.5)
**Twin**	
Yes (twin)	28 (3.3)
No (singleton)	815 (96.7)
**Major congenital malformation**	
Yes	9 (1.1)
No	832 (98.7)

* *Mother age (N = 824), birth weight (N = 841), gestational age (N = 840), Husband's occupation (N = 826), Number of wives (N = 804), Number of children (N = 826), education (N = 822), Occupation (N = 826), ethnicity (N = 823)*.

### Stillbirths

Thirteen stillbirths (15.4 per 1,000 births 95% CI 7.1–23.8) were reported during the study. Nine of these were fresh stillbirths and the remaining four were macerated stillbirths. The causes attributed to the fresh stillbirths were poor monitoring of labor (*n* = 3), twin gestation (*n* = 2), poor progression of labor (*n* = 2), eclampsia (*n* = 1) and Down syndrome (*n* = 1). Six out of the nine fresh stillbirths occurred during night shifts (from 8 p.m. to 8 a.m.).

### Neonatal Hospitalizations and Associated Risk Factors

**Figure 1:**
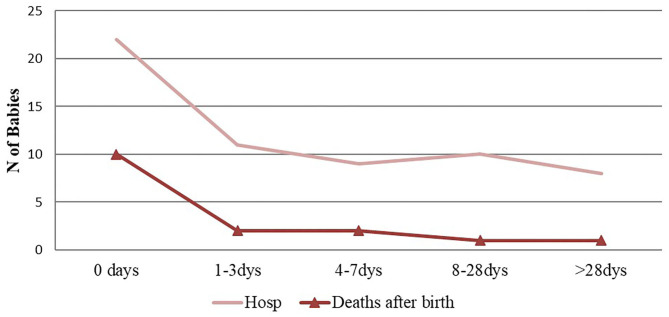
Pattern of hospitalization and deaths during the study period (0–56) days.

**Figure 2:**
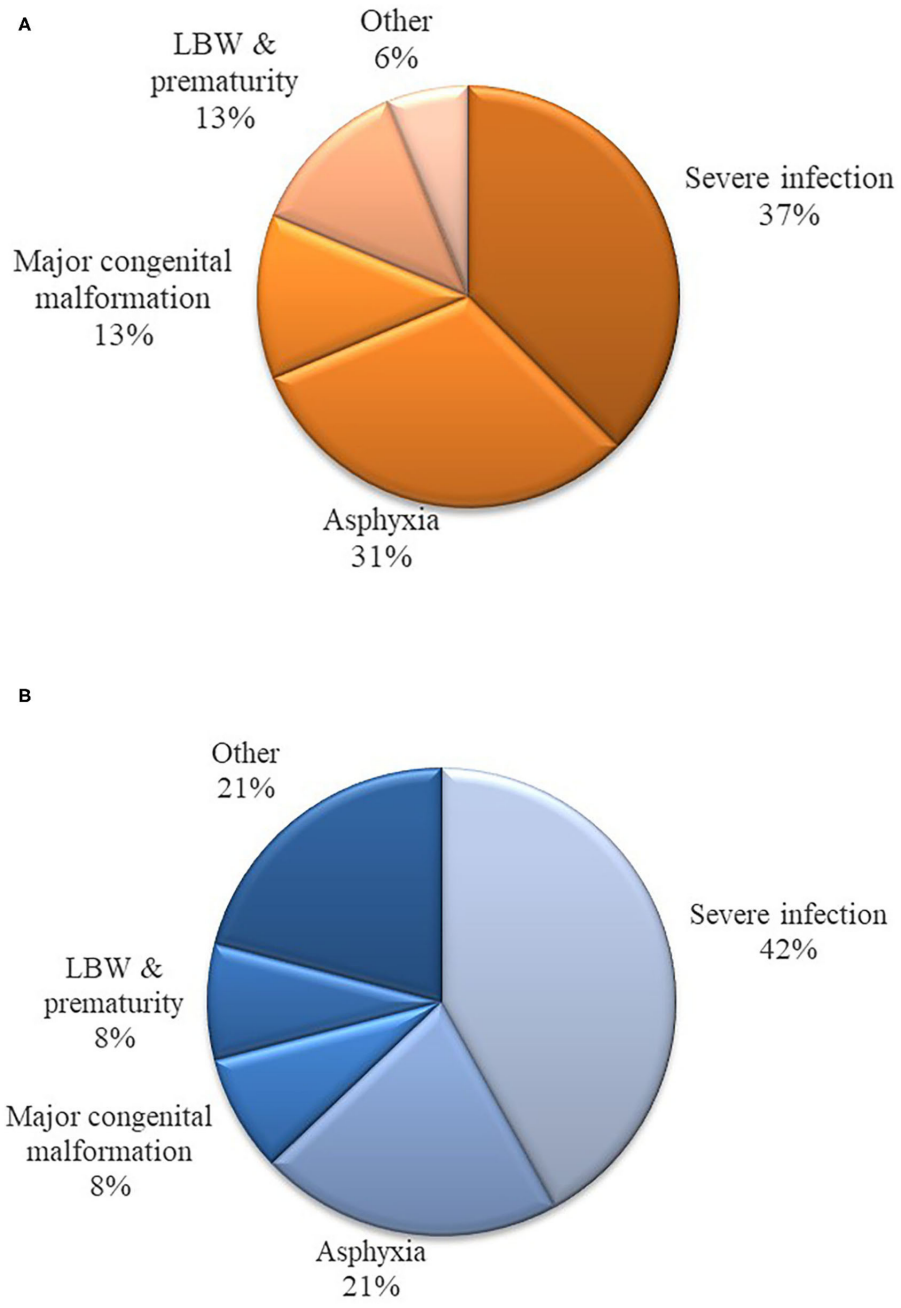
**(A)** Main diagnoses at hospitalization during the study period (0–56) days. **(B)** Main diagnoses of death during the study period (0–56) days.

Out of 829 live-births, 63 children (7.6%) were hospitalized during the 8-week follow-up period. Seventy-five percent of hospitalized infants were admitted during the early neonatal period (Figure 1). Most who were hospitalized had multiple diagnoses at admission. The most common diagnosis was severe infection (i.e., sepsis, meningitis and pneumonia) (*n* = 27), followed by birth asphyxia (*n* = 13), jaundice (*n* = 6), and low birth weight (*n* = 5). Nine newborns were hospitalized with major congenital malformations, and five of these had congenital malformation as the main diagnosis at admission (Figure 2A). Four of the nine babies had major congenital heart defects, three co-existing with Down Syndrome and one baby diagnosed with talipes equinovarus. Three babies had isolated talipes equinovarus, one baby had a cleft lip and palate, one had choanal atresia and one had encephalocele.

During the study period, we observed two clusters of hospitalizations. One cluster included six cases of clinical sepsis (27.3% of overall sepsis; none confirmed microbiologically) in June 2013. The second cluster, between January and April 2014, included 10 cases of severe birth asphyxias, 76.9 % of total cases (*n* = 13) reported in the study.

In the multivariate analysis, low birth weight, major congenital malformations, twin births and cesarean section deliveries remained significantly associated with hospitalization (Table 2).

**Table 2:** Risk factor analysis for hospitalization during the study period (0–56 days) (*N* = 829 live births).

	**Hospitalization**	**Univariable**		**Multivariable**^****1****^	
	***n* (%)**	**OR (95%CI)**	***p*-value**	**OR (95%CI)**	***p*-value**
**Sex**					
Male	31 (7.19)		0.653		0.986
Female	32 (8.02)	1.13 (0.67–1.88)		0.99 (0.57–1.75)	
**Ethnicity**					
Mandinka	29 (8.43)				
Wollof	5 (5.38)	0.62 (0.23–1.64)	0.333		
Jola	8 (6.35)	0.74 (0.33–1.66)	0.46		
Fula	9 (6.38)	0.74 (0.34–1.61)	0.448		
Others	11 (10.38)	1.26 (0.61–2.61)	0.539		
**Birth weight (gram)**					
Normal (≥2,500)	50 (6.44)		<0.001	2.91 (1.24–6.82)	0.014
Low (<2,500)	13 (24.53)	4.72 (2.37–9.39)			
**Major Congenital malformation**					
No	58 (7.13)	21.95 (5.12–94.16)	<0.001	24.27 (5.23–112.71)	<0.001
Yes	5 (62.5)				
**Twin**					
No	52 (6.46)		<0.001		<0.001
Yes	11 (44)	11.38 (4.92–26.31)		8.20 (3.00–22.39)	
**Season of delivery**					
Rainy season	15 (8.57)		0.378		
Early dry	10 (6.06)	0.69 (0.3–1.58)	0.732		
Late dry	38 (7.76)	0.9 (0.48–1.67)			
**Mode of delivery**					
Vaginal	59 (7.22)		0.005		0.005
Cesarean section	4 (30.77)	5.71 (1.71–19.1)		7.05 (1.80–27.49)	
**Maternal age in years**					
=<19	1 (1.52)	0.18 (0.02–1.33)	0.093	0.79 (0.32–1.97)	0.617
20–34	55 (7.83)	1 (reference)			
≥35	7 (12.28)	1.65 (0.71–3.81)	0.243	1.79 (0.61–5.24)	0.286
**Number of children**					
1	15 (8.62)		0.743		
2–3	26 (7.78)	0.89 (0.46–1.74)	0.489		
≥4	21 (6.89)	0.78 (0.39–1.56)			
**Number of father's wives**					
1	44 (7.09)	1 (reference)	0.209		
>2	17 (10.0)	1.46 (0.81–2.62)			
**Father employment**					
None/Home	3 (7.5)		0.915		
UnSkilled	16 (8)	1.07 (0.3–3.87)	0.631		
Skilled	20 (5.62)	0.73 (0.21–2.59)	0.698		
Business	7 (5.79)	0.76 (0.19–3.08)	0.165		
Others	16 (16.84)	2.5 (0.69–9.11)			
**Mother employed**					
No	45 (6.56)	1 (reference)	0.009		0.067
Yes	17 (13.39)	2.20 (1.22–3.99)		1.89 (0.96–3.75)	
**Mother's education in years**					
<6	29 (6.32)				0.098
≥6	33 (10.06)	1.66 (0.99–2.79)	0.057	1.66 (0.91–3.02)	
Unknown	0 (0)	–			

1 *Adjusted for sex, low birth weight, major congenital malformation, twin births, mode of delivery, maternal age, mother education level and occupation*.

### Neonatal Mortality

Sixteen deaths occurred during the follow up period. Almost two thirds (62.5%) of these deaths occurred during the early neonatal period (Figure 1). Only one death occurred after the first 28 days of life. Overall, perinatal mortality rate (PMR) and neonatal mortality rate (NMR) were 26.1 (95%CI: 15.3–36.9) and 19.3 (95%CI 9.9–28.7) per 1,000 live births, respectively. The distribution of cause-of-death during the neonatal period is shown in Figure 2B. In the multivariate analysis, major congenital malformations and twin deliveries were the main risk factors for neonatal mortality (Table 3).

**Table 3:** Risk factors of death during the study period (0–56 days) (*N* = 829 live births).

		**Univariable**		**Multivariable**^****1****^		
	**Total**	**Neonatal mortality**	**OR (95%CI)**	***p*-value**	**OR (95%CI)**	***p*-value**
		***n* (%)**				
	**830**	**16 (1.9)**				
**Sex**						
Male	431	9 (2.1)	1 (reference)	0.727	1 (reference)	0.478
Female	399	7 (1.7)	0.84 (0.31–2.27)		0.68 (0.24–1.96)	
**Ethnicity**						
Mandinka	344	9 (2.6)	1 (reference)			
Wollof	93	1 (1.1)	0.4 (0.05–3.23) 0.32 (0.07–1.38)	0.394		
Jola	126	1 (0.8)	0.3 (0.04–2.37)	0.253		
Fula	141	2 (1.4)	0.54 (0.11–2.51)	0.428		
Others	106	2 (1.9)	0.72 (0.15–3.37)	0.672		
**Birth weight (grams)**						
Normal (≥2,500)	776	13 (1.7)	1 (reference)	0.055	1 (reference)	0.612
Low (<2,500)	53	3 (5.5)	3.52 (0.97–12.76)		1.52 (0.30–7.66)	
**Major congenital malformation**						
No	809	13 (1.6)	1 (reference)	<0.001	1 (reference)	<0.001
Yes	8	3 (37.5)	26.6 (6.18–114.48)		36.15 (7.82–167.04)	
**Twin**						
No	805	13 (1.6)	1 (reference)	0.002	1 (reference)	0.008
Yes	25	3 (10.7)	8.31 (2.21–31.25)		8.81 (1.74–44.53)	
**Season of delivery**						
Rainy season	175	1 (0.6)	1 (reference)			
Early Dry season	165	2 (1.2)	2.13 (0.19–23.77)	0.537		
Late Dry season	490,175	13 (2.6)	4.74 (0.62–36.52)	0.135		
	165					
	490					
**Mode of delivery**						
Vaginal	817	16 (1.9)			5.45 (1.06–28.45)	
Cesarean section	13	0 (0)				
**Maternal age**						
≤19	0	0 (0)	–			
20–34	702	15 (1.8)	1 (reference)			
≥35	57	1 (5.2)	2.94 (0.81–10.65)	0.100		
**Number of children**						
1	174	2 (1.1)	1 (reference)			
2–3	334	5 (1.5)	1.31 (0.25–6.81)	0.750		
≥4	305	8 (2.6)	2.32 (0.49–11.03)1.08 (0.42–2.75)	0.29		
**Mother employed?**						
No	686	12 (1.7)	1 (reference)	0.639		
Yes	127	3 (2.3)	1.35 (0.38–4.88)			
**Mother's education in yrs**						
<6	459	7 (1.5)	1 (reference)	0.36		
≥6	328	8 (2.4)	1.61 (0.58–4.5)			
Unknown	22	0 (0)	–			

1 *Adjusted for sex, low birth weight, major congenital malformation, and twin births*.

## Discussion

We presented hospital-based data of hospitalization and deaths during the neonatal period in an urban health facility in Western Gambia from women without known risk factors due to the exclusion criteria of the trial. Our results show high rates of poor neonatal pregnancy outcomes, such as stillbirths, neonatal hospitalization, and mortality. The causes of perinatal death varied, but stillbirths, severe clinical infections, birth asphyxia and major congenital malformations were the most common; often, these conditions could have been prevented or mitigated by early identification and preventative measures. However, the busy public urban health facility where the study took place lacked material resources, skilled capacity, and space to effectively manage deliveries. Conversely, there were no maternal deaths out of the 829 women in the study, which, when considered against the mortality rate previously described in rural Gambia (461 per 100,000 live births) (22), four deaths would have been anticipated.

Stillbirths represented approximately half of all deaths observed in the study; most of the observed cases were fresh stillbirths. The number of macerated stillbirths is likely underestimated as confirmed intra-uterine fetal death was an exclusion criterion for women prior to recruitment in the trial (16). The facility also lacked the capacity to determine if any of these cases had an underlying congenital malformation, prenatally. Most stillbirths reported during the trial occurred during night shifts when deliveries were attended to by fewer trained and skilled staff e.g., non-midwife staff or trainee nursing students on training placements. Therefore, this high number of fresh stillbirths was at least in part attributable to delivery attendance by non-skilled health care staff, poor labor monitoring and delay in early diagnosis of obstetric emergencies, as well as late referral for emergency obstetric care (23). Despite the high rate of stillbirths reported in this study, this rate has significantly decreased compared to results from a hospital-based study conducted in The Gambia in 1988. Late referrals were a main risk factor for stillbirths in the former study, indicating that some gaps in the health system have remained problematic for several decades (23).

Two clusters of hospitalizations, one of birth asphyxia and the other of neonatal sepsis, further demonstrate the impact of gaps in the health system on poor pregnancy outcomes. Birth asphyxia was the second most common cause of hospitalization and neonatal deaths. Severe birth asphyxia may be prevented by early identification; however, delay in detection and subsequent non-skilled birth attendance often culminated in serious intrapartum events that result in cases of birth asphyxia. Almost 80% of these cases observed during this trial occurred during this cluster, which coincided with the absence of an experienced night-shift midwife during peak months of delivery. This indicates that the absence of trained healthcare personnel may have had an impact on outcomes for these cases, a common situation in many health facilities across The Gambia and SSA (24, 25). The sepsis cluster coincided with a dysfunctional autoclave used for sterilization of delivery kits, as well as poor hygienic states in both the delivery and post-natal wards. During this instance, six cases of neonatal sepsis were reported within 4 weeks. When the autoclave was identified as dysfunctional and was repaired, along with the introduction of a weekly general ward cleaning exercise, the cases of neonatal sepsis drastically reduced.

Multiple pregnancy was also a risk factor for poor pregnancy outcomes; twins accounted for 18.8% of neonatal deaths in this study, and 22.2% of fresh stillbirths. These data are consistent with a community-based analysis conducted as part of the Health and Demographic Surveillance System in rural Gambia where ~1 in 8 neonatal deaths occurred among twins (9). To improve outcomes for multiple pregnancies, early detection and referral is vital; again, this indicates the importance of attendance to deliveries by skilled healthcare personnel.

Major congenital malformations was another risk factor for both hospitalization and mortality, representing 18.8% of our neonatal deaths. According to estimates generated by the WHO and Maternal and Child Epidemiology Estimation Group (MCEE) 2018, congenital abnormalities was the fourth leading cause of neonatal deaths in The Gambia in 2015 accounting for up to 10% all neonatal deaths (26). The higher prevalence found among fatalities in our study may be linked to the study design, where all our participants were evaluated by a research clinician hours after birth and therefore any congenital malformations were more likely to be detected. Also, the high burden of major congenital malformations in The Gambia is not surprising given the high frequency of consanguineous marriages (~30% among first-cousin marriages) (17), a described risk factor in countries where this practice is common (27). In a retrospective study in Tunisia, where rates of consanguinity are higher than in The Gambia (40–49%) (28), almost half of autopsies showed a congenital malformation in the newborn, and parental consanguinity was identified as a main risk factor (29). Prospective studies conducted in high-income countries have also shown that congenital malformations are a major cause of stillbirth and neonatal mortality; it is reasonable to assume that congenital malformations cause an even greater number of stillbirths and neonatal deaths in low-income settings like The Gambia (30) due to the lack of necessary equipment and capacity available to detect these malformations, combined with limited access to preventive care, skilled healthcare workers, and emergency obstetric facilities (24).

A recent hospital-based neonatal mortality audit conducted in the main referral hospital in The Gambia, showed that low birth weight was an independent predictor of neonatal death (31). We found the same association for hospitalization although the trend is not significant for mortality, probably due to lack of power or the correlation between low-birth weight and twin births.

Our health facility-based study complements previous data generated from community-based studies as more than 80% of deliveries in The Gambia take place in health facilities (14). In addition, ours uses data that was collected prospectively in contrast to former retrospective reviews of inpatient data (23, 31). The main limitation of our analysis, however, is that it uses data from a single health facility which may not be representative of all facilities in The Gambia as wide variability between health facilities have been described in the region (7, 14, 32). On the other hand, the care for the participants in this study was likely above national standards. The intense follow-up of study participants probably led to earlier detection, improved clinical care and increased hospitalization rates; but might have reduced mortality and skewed the diagnosis as most deaths due to infections that were more promptly diagnosed and quickly treated probably improved outcome. After labor, each participant was discharged from the health facility by a clinician or pediatrician, which should have resulted in more accurate diagnoses of congenital malformations which may otherwise have been missed. The study criteria also potentially affected outcomes: the recruited women excluded those with high risk pregnancies, as well as participants with chronic conditions who required on-going medication. For instance, even the high rate of stillbirths and neonatal deaths reported were approximately half the rates described by United Nations and WHO 2017 countdown to 2030 progress report (24 per 1,000 total births and 28 per 1,000 live births, respectively, in the year 2015) (26). The results of this analysis may further underestimate overall rates of severe morbidity and mortality due to severe infections; half of the newborns in the study were born to mothers who received azithromycin during labor. We observed statistically significant differences between trial arms in less-severe outcomes, although the trial was not designed or powered to detect differences in mortality. Additionally, in the analysis of arms comparison, we found that deaths in the intervention arm but not in the placebo arm were associated with an underlying condition (15).

In conclusion, the results of this analysis indicate rates of neonatal hospitalization and mortality in The Gambia are still high. Although vertical interventions may reduce specific causes of death, improving weak health systems is a critical factor in widening the impact of such interventions to reach targets set in the Sustainable Development Goal 3.2. There should also be emphasis on investigating ways to reduce complications during labor which result in fresh stillbirth and severe birth asphyxia. Initiatives must be undertaken to train healthcare workers on the importance of preventive care including infection control and early identification of risk factors. Though vertical interventions present opportunities for impact on specific causes of neonatal morbidity and mortality, the quality of a health system may ultimately determine outcomes for this vulnerable population; further investigations are warranted to identify high-risk gaps in health systems during delivery and postnatal care.

## Data Availability Statement

The raw data supporting the conclusions of this article will be made available by the authors, without undue reservation.

## Ethics Statement

The studies involving human participants were reviewed and approved by Joint Gambia Government/MRC Ethics Committee. Written informed consent to participate in this study was provided by the participants' legal guardian/next of kin.

## Author Contributions

BC developed and adapted the field work and contributed to collection of data in the main trial, interpreted data and wrote the initial manuscript. CO developed and adapted the field work and contributed to collection of data in the main trial and made contributions to the manuscript. RM analyzed the data and made contributions to the manuscript. UO contributed significantly to the manuscript. AL contributed to the revision of the manuscript. BK contributed to the protocol of the main trial and contributed significantly to the manuscript. KM contributed to the implementation of the main trial and revised the manuscript. UD'A conceived and contributed significantly to the final version of the study design and protocol and critically revised the manuscript. AR conceived and designed the study, drafted the protocol, and substantially contributed to the statistical analysis and writing of the manuscript. All authors contributed to the article and approved the submitted version.

## Conflict of Interest

The authors declare that the research was conducted in the absence of any commercial or financial relationships that could be construed as a potential conflict of interest.
